# Effect of Ultrasound-Assisted Extraction and Drying Methods on Bioactive Compounds, Phenolic Composition, and Antioxidant Activity of Assam Tea Cultivar (*Camellia sinensis* var. *assamica*) Cultivated in Thailand

**DOI:** 10.1155/2024/5772961

**Published:** 2024-10-30

**Authors:** Sakan Warinhomhoun, Jiraporn Raiputta, Paryn Na Rangsee, Chung S. Yang, Piyaporn Chueamchaitrakun

**Affiliations:** ^1^College of Oriental Medicine, Rangsit University, Lak Hok 12000, Pathum Thani, Thailand; ^2^School of Medicine, Walailak University, Nakhon Si Thammarat 80160, Thailand; ^3^Center of Excellent in Marijuana, Hemp and Kratom, Walailak University, Nakhon Si Thammarat 80160, Thailand; ^4^Tea and Coffee Institute, Mae Fah Luang University, Chiang Rai 57100, Thailand; ^5^Department of Chemical Biology, Rutgers University, 164 Frelinghuysen Road, Piscataway 08854, New Jersey, USA; ^6^School of Agro-Industry, Mae Fah Luang University, Chiang Rai 57100, Thailand

**Keywords:** antioxidant activity, *Camellia sinensis* var. *assamica*, catechins, freeze drying, spray drying, Thai Assam tea, ultrasound-assisted extraction

## Abstract

Tea is a rich source of phytochemicals; their composition in tea extracts varies depending on the cultivar, climate, production region, and processing and handling processes. The method of extraction plays a crucial role in determining the biological effects of the bioactive compounds in tea leaves. However, reports on the catechin profiles and antioxidant activities of the extracts obtained from leaves at different stages of maturity are limited. Here, we aimed to evaluate the effect of ultrasound-assisted extraction (UAE) and different drying methods, freeze drying (FD) and spray drying (SD), on the composition of bioactive compounds, phenolic composition, and antioxidant activity of extracts obtained from different part of leaves, top (TT), middle (ML), and mature (MT), of Assam tea cultivar (*Camellia sinensis* var. *assamica*) cultivated in Thailand (Thai Assam tea). High-performance liquid chromatography analysis showed that the extracts obtained by UAE with FD from TT leaves (UAEFD-TT) had the highest catechins (341.38 ± 0.11 mg/g extract) and caffeine (93.20 ± 0.36 mg CF/g extract) contents compared with those extracted from ML and MT using the same method as well those obtained by SD. The total phenolic and total flavonoid contents were the highest in UAEFD-TT extracts (456.78 ± 4.31 mg GAE/g extract and 333.98 ± 0.83 mg QE/g extract, respectively). In addition, UAEFD-TT exhibited the highest antioxidant activity; the IC_50_ values obtained by 2,2-diphenyl-1-picrylhydrazyl (DPPH) and 2,2′-azino-bis (3-ethylbenzothiazoline-6-sulfonic acid) (ABTS) assays were 1.31 ± 0.02 and 7.51 ± 0.03 *μ*g/mL, respectively. In the ferric-reducing antioxidant power (FRAP) assay, the UAEFD-TT extract demonstrated the highest antioxidant activity (324.54 ± 3.33 *μ*M FeSO_4_/mg extract). These results suggest that extraction from TT using UAE followed by FD produced the highest amount of antioxidant compounds in Thai Assam tea extracts.

## 1. Introduction

Tea, made by steeping processed leaves, buds, or twigs of the tea plant (*Camellia sinensis*) in hot water, is a widely consumed beverage globally. Tea is known for its various flavors, aromas, and health benefits and is consumed in several different forms, such as green, black, or oolong tea. Green tea leaves predominantly contain the catechin epigallocatechin gallate (EGCG) (40%–69%), followed by epigallocatechin (EGC) (12%–23%), epicatechin gallate (ECG) (13%–21%), and epicatechin (EC) (5%–9%) [1–3]. Nonetheless, these ratios may differ depending on factors such as environmental conditions, type of tea, and agricultural methods such as harvest timing and the age of the leaves [4, 5]. In addition, younger leaves contained greater amounts of EGCG and ECG when compared to older leaves, while the mature (MT) leaves exhibited higher levels of EGC and EC. This suggests that the catechin profiles in green tea extracts (GTEs) can differ based on the origin, age of the tea leaves, and the method of extraction employed, which may influence their antioxidant properties [6, 7]. Tea polyphenols, commonly known as catechins, are unique constituents of tea and account for 30%–42% of the soluble ingredients in tea. Green tea has been reported to exhibit numerous health benefits [8–10]. The antioxidant activities of catechins are closely related to their structure. For instance, hydroxyl groups at Positions 5 and 7 of the A-ring, an ortho-3′4′-dihydroxyl group (catechol) or 3′4′5′-trihydroxyl group (pyrogallol) in the B-ring, and a gallate group located at Position 3 of the C-ring contribute to the antioxidant properties of polyphenolic compounds [11]. Preclinical and human studies have revealed the antioxidant, antimicrobial [12–14], antidiabetes [14], [15–17], anti-*β*-amyloid [18], anticancer [14, 19], anti-inflammation [20, 21], antiallergy [22, 23], and antiviral effects of catechins [24–26]. Despite these health benefits, catechins are also the major contributors to the astringency and bitterness of green tea infusions [15, 27]. These properties of the catechins are influenced by several factors, such as molecular size, chemical structure, and interactions with other compounds. For instance, catechins have a large molecular size and comprise many hydrogen bonds, which affects their bioavailability. Furthermore, the varied behaviors of the catechins, including their bioactivity and intermolecular interactions, are attributed to the differences in their steric configuration and molecular structure of catechins [28]. In addition, the extraction plays a crucial role in determining the biological effects of the bioactive compounds in tea leaves.

Different methods such as solvent extraction, mechanical expelling, supercritical fluid extraction, microwave-assisted extraction, and ultrasound-assisted extraction (UAE) are employed for the recovery of bioactive compounds in tea leaves. Among these methods, UAE is preferred over others owing to their potential limitations, such as the use of extra solvent in solvent extraction, low yield in mechanical expelling, large capital in supercritical fluid extraction, and the requirement of an aqueous phase in microwave-assisted extraction. Furthermore, high-temperature extraction often leads to the degradation of polyphenols as well as increased protein and pectin extraction, which interfere with the organoleptic quality of tea by cream formation. In contrast, the UAE has advantages such as reduced time and energy requirements, extraction at low temperatures, and retention of extract quality [29, 30]. In this process, the mechanical and thermal effects of high-frequency sound waves are used to disrupt cell tissues to release the bioactive compounds, which facilitate the efficient extraction of plant materials [31], including phenolic and flavonoid compounds [30, 32–35]. This method has gained popularity in various industries, particularly the food and pharmaceutical industries [36]. UAE is also widely used in tea leaf extraction and has been shown to increase polyphenol yield at a low temperature (65°C) compared with that at high temperature (85°C) [37–39].

The drying process significantly affects the quality of GTE, as it can impact the concentration of bioactive compounds and the overall quality of the extract. Two common methods used for drying GTE are spray drying (SD) and freeze drying (FD). Studies have demonstrated that SD is a widely used method for drying GTE due to its efficiency and cost-effectiveness [40–42]. While FD is particularly favored for its ability to retain the bioactive compounds [43–45] by preventing the thermal degradation of temperature-sensitive compounds [46] and preserving sensory attributes of the extract [47].

In Thailand, two major tea varieties, Assam tea (*Camellia sinensis* var. *assamica*) and Chinese tea (*C. sinensis* var. *sinensis*), are cultivated. Among them, Assam tea (hereinafter referred to as Thai Assam tea) is the major tea variety and is cultivated in a larger planting area than Chinese tea [48]. Thai Assam tea, particularly in its black tea form, is one of the most widely consumed beverages in Thailand. In addition, Thai Assam tea is used in northern Thailand to produce traditional fermented chewing snack products, such as Miang [49]. Studies have reported that Thai Assam tea exerts antioxidant [50–55], antimicrobial [51, 52], anti-inflammatory [52, 55], alpha-amylase [52], alpha-glucosidase [52], and anticancer [51, 56, 57] activities. However, reports on the catechin profiles and antioxidant activities of the extracts obtained from leaves at different stages of maturity are limited. Therefore, in this study, we aimed to determine the effects of UAE extraction followed by drying using different methods, such as FD and SD, on the catechin contents of the extracts obtained from the different ages of tea leaves: top (TT), middle (ML), and MT leaves using UAE. In addition, we assessed the antioxidant properties of all Thai Assam tea extracts.

## 2. Materials and Methods

### 2.1. Sample Collection and Processing

**Figure 1:**
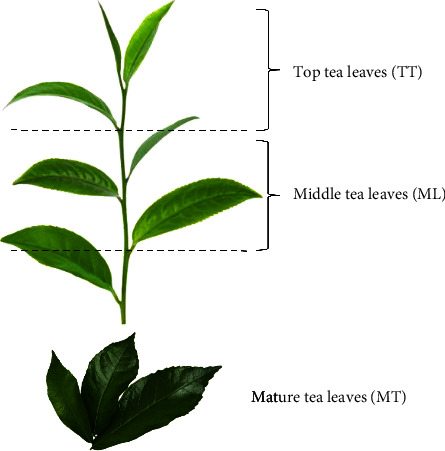
Different parts of Thai Assam tea used for sample preparation in this study.

Thai Assam tea leaves were obtained from the Wawee tea plantation (Chiang Rai, Thailand) in 2023. The top leaves were carefully harvested, then withered and dried at 60°C for 120 min, reducing the moisture content to approximately 5%, This process followed a method similar to green tea production, which involves no oxidation and no fermentation. The leaves were placed in a sealed plastic bag and immediately transferred to the Tea and Coffee Institute's Laboratory, Mae Fah Luang University (Chiang Rai, Thailand). The dried tea leaves were categorized into three age groups: TT, comprising the first and second leaves after the bud; ML, comprising the third to sixth leaves; and MT, comprising the seventh and older leaves (Figure 1). The samples from the three different ages were then ground separately into powder forms (particle size of approximately 150 *μ*m) using a commercial laboratory blender homogenizer (51BL30, Waring Commercial, USA). All samples were placed in plastic bags and stored at ambient temperature until extraction.

### 2.2. Preparation of Thai Assam Tea Extract

#### 2.2.1. UAE

Approximately 100 g ground samples (TT, ML, and MT) were mixed with 50% ethanol (%v/v) at a fixed solid/liquid ratio of 1:20 (%w/v) [58]. The extraction was carried out in an ultrasonic water bath (Elmasonic S100; Elma Schmidbauer GmbH, Singen, Germany). The UAE was performed under the following conditions: 30°C and operated at 37 kHz frequency for 20 min [33]. The extracts were then filtered using a Whatman® No. 1 filter paper. Each filtrate was concentrated to dryness in a rotary evaporator (Heidolph Instruments GmbH & Co., Germany) at a controlled temperature (50°C) to obtain the final crude extracts, which were then stored at 4°C in a refrigerator until further use (Table S1).

#### 2.2.2. SD and FD

SD was carried out using a spray dryer (Büchi, Flawil, Switzerland). A two-fluid nozzle with a capacitive diameter of 0.5 mm was used. The operating conditions were as follows: drying air inlet temperature, 200°C–350°C; atomization air volumetric flowrate, 400 L/h; feed volumetric flow rate, 3 mL/min; and drying air volumetric flow rate, 35–38 m^3^/h. The air outlet temperature (80°C) was measured for each extract, and the final extract was stored at −30°C in a refrigerator until further use (Table S1).

For FD, the crude extracts obtained in Section 2.1.1 were frozen at −40°C for 48 h and placed in a freeze dryer equipped with a capacitance manometer to monitor the condenser pressure for 48 h under vacuum (0.1–0.01 mmHg) until a constant moisture content was achieved. The chamber and ice condenser temperatures were set at −45°C to obtain the final extracts, which were then stored at −30°C in a refrigerator until further use (Table S1).

### 2.3. Determination of Contents of Catechin Derivatives and Caffeine (CF)

The final extracts (0.1 g) obtained in Section 2.2 (UAESD-TT, UAESD-ML, UAESD-MT, UAEFD-TT, UAEFD-ML, and UAEFD-MT) were dissolved in 100 mL stabilizing solution (10% acetonitrile with 500 *μ*g/mL ethylenediaminetetraacetic acid (EDTA) and 500 *μ*g/mL ascorbic acid) at a concentration of 1 mg/mL. The solution was then filtered through a 0.22 *μ*m nylon membrane filter and used as the sample for HPLC analysis.

The contents of catechin derivatives and CF were quantified using HPLC, as described in a previous study [59]. HPLC was performed using a Nexera LC-40B xR system consisting of an autosampler (Shimadzu, Japan), a column oven (Shimadzu, Japan), and a photodiode array detector (Shimadzu, Japan). Liquid chromatography was performed using a Platinum C18-EPS column (53 × 7 mm, 3 *μ*m) controlled at 35°C with isocratic elution at a flow rate of 1 mL/min. The mobile phase comprised acetonitrile and 0.05% TFA at a ratio of 13:87 (v/v). The injection volume was 10 *μ*L, and the total run time was 10 min. The measurement wavelength was set at 210 nm. The standard curve developed using catechin derivatives and CF purchased from Sigma-Aldrich (USA) was used to quantify their contents in the extracts. The retention times of the reference standards and the tea extracts containing various components were as follows: gallocatechin, GC 1.271 min; EGC 1.668 min; catechin, C 1.937 min; EC 2.563 min; EGCG 2.828 min; CF 3.465 min; gallocatechin gallate (GCG) 3.906 min; ECG 4.631 min; and catechin gallate (CG) 6.221 min, respectively (Table S2).

### 2.4. Determination of Total Phenolic Content (TPC)

The TPC of the samples was determined according to the International Standards Organization (ISO) 14502-1-2005 E procedure using the Folin–Ciocalteu reagent (Merck, Darmstadt, Germany). In brief, 1 mL extract was mixed with 5 mL of 10% diluted Folin–Ciocalteu's reagent and 4 mL of 7.5% Na_2_CO_3_. The mixture was then incubated at room temperature for 1 h, and the absorbance was measured at 765 nm using a UV–visible spectrophotometer (Thermo Fisher Scientific, Madison, Wisconsin, USA). All samples were analyzed in triplicate. Gallic acid (GAE) (10–100 *μ*g/mL; Sigma-Aldrich) was used as a positive control. The results were expressed as milligrams of GAE per gram of extract (mg GAE/g extract).

### 2.5. Total Flavonoid Content (TFC)

The TFC was determined using an aluminum chloride colorimetric assay, as described in a previous study [60], with some modifications. In brief, 5 mL extract was mixed with 10 *μ*L of 10% aluminum chloride (Sigma-Aldrich) and 2.0 mL NaOH solution. The mixture was then incubated in the dark at room temperature for 40 min. Absorbance was measured at 510 nm using a UV–visible spectrophotometer (Thermo Fisher Scientific). All samples were analyzed in triplicates. A solution of quercetin (QE) (0–12 mg/mL; lot number: Q4951; Sigma-Aldrich) was used to prepare a standard curve to determine the TFC of the extracts. The results were expressed as milligrams of QE equivalents per gram of extract (mg QE/g extract).

### 2.6. Determination of Free Radical Scavenging Activities

The free radical scavenging activity of Thai Assam tea extract was determined using the 2,2-diphenyl-1-picrylhydrazyl (DPPH) assay [61]. In brief, the sample (50–500 *μ*g/mL) was added to 6.0 × 10^−5^ mol/L DPPH solution in a 96-well plate. The mixture was then incubated for 30 min at room temperature in the dark. Absorbance was measured at 517 nm using a microplate reader (Thermo Fisher Scientific, Göteborg, Sweden). EGCG concentration at 0.625–10 *μ*g/mL was used as a positive control. The percent scavenging activity (%SA) was calculated by using the following equation:

(1) $$\begin{array}{c} \%\text{SA}=\left[\frac{\left(A_{\text{sample}}-A_{\text{blank}}\right)}{A_{\text{blank}}}\right]\times 100, \end{array}$$

where *A*_sample_ is the absorbance of the DPPH-treated sample at 517 nm and *A*_blank_ is the absorbance of DPPH-treated methanol at 517 nm.

The 2,2′-azino-bis (3-ethylbenzothiazoline-6-sulfonic acid) (ABTS) assay was conducted according to a previously described method with some modifications [62]. In brief, 7 mM ABTS (36.0 mg) was dissolved in 10 mL K_2_S_2_O_8_ (2.45 mM), and the solution was incubated at room temperature for 18 h in the dark, resulting in the generation of ABTS^+^ radicals. After 18 h, the ABTS solution was diluted with methanol to ensure a consistent absorbance of 0.70 ± 0.02 at 734 nm. The sample (20 *μ*L) at 1.25–20 *μ*g/mL was mixed with the solution containing ABTS^+^ radicals (180 *μ*L) in a 96-well plate. The mixture was then incubated for 30 min at room temperature in the dark. Absorbance was measured at 734 nm using a microplate reader (Thermo Fisher Scientific). EGCG at a concentration between 1 and 10 *μ*g/mL was used as a positive control. The %SA was calculated by using the following equation:

(2) $$\begin{array}{c} \%\text{SA}=\left[\frac{\left(A_{\text{sample}}-A_{\text{blank}}\right)}{A_{\text{blank}}}\right]\times 100, \end{array}$$

where A_sample_ is the absorbance of the sample treated with ABTS at 734 nm and A_blank_ is the absorbance of ABTS-treated methanol at 734 nm.

The 50% inhibitory concentration (IC_50_) values of the samples in both DPPH and ABTS assays were determined from a graph plotted against the concentration and percentage of inhibition.

### 2.7. Ferric-Reducing Antioxidant Power (FRAP) Assay

The ability of the tea extracts to chelate ferrous ions was evaluated using a colorimetric method described in a previous study with some modifications [62]. The FRAP reagent was prepared by mixing 2.5 mL of 10 mM TPTZ stock solution, 25 mL acetate buffer (300 mM, pH 3.6), and 2.5 mL 20 mM FeCl_3_ solution (1:10:1). Then, 50 *μ*L of the sample (0.5 mg/mL) was mixed with 150 *μ*L FRAP reagent in a 96-well plate. The mixture was then incubated for 30 min at room temperature in the dark. The absorbance of the intense blue complex formed after the incubation was measured at 594 nm using a microplate reader (Thermo Fisher Scientific), and the reducing capacity was expressed as *μ*M FeSO_4_/mg extract.

### 2.8. Statistical Analysis

All data were obtained from three dependent experiments and are expressed as the mean ± standard deviation (SD). Statistical analyses were performed using GraphPad Prism 10.0.3 (GraphPad Software Inc., San Diego, California, USA) using one-way analysis of variance (ANOVA). Differences at *p* < 0.05 were considered statistically significant.

## 3. Results

### 3.1. Identification and Quantitative Analysis of Thai Assam Tea Extracts

**Figure 2:**
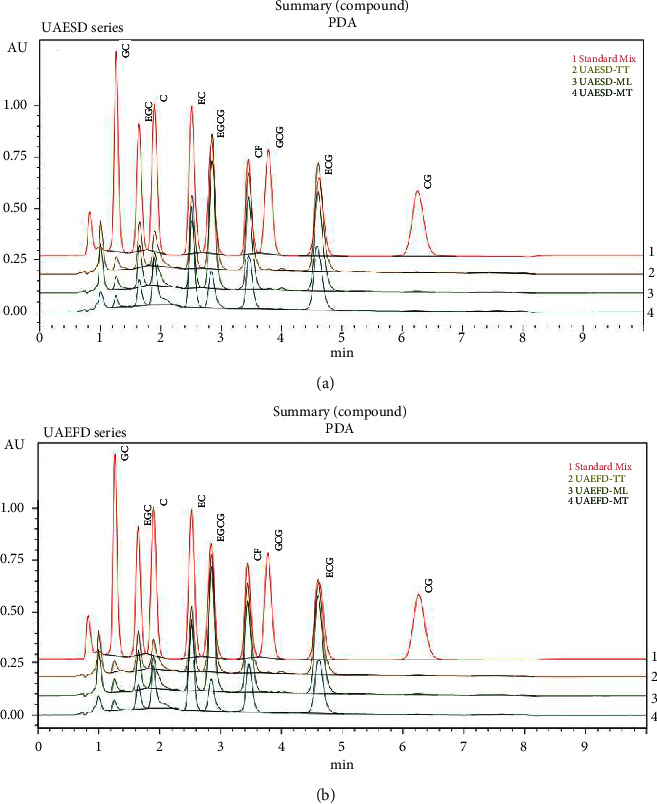
HPLC chromatogram of standard mix of catechin, caffeine (CF: caffeine, C: catechin, CG: catechin gallate, EC: epicatechin, ECG: epicatechin gallate, EGC: epigallocatechin, EGCG: epigallocatechin gallate, GC: gallocatechin, and GCG: gallocatechin gallate), and Thai Assam tea extracted with 50% ethanol UAE and drying method using spray drying (a) and freeze drying (b) with wavelength of 210 nm, flowrate of 1 mL/min, and injection volume of 10 *μ*L.

The chromatographic elution of different catechin derivatives and CF in the final extracts of Thai Assam tea is shown in Figure 2. The catechin derivative contents in all extracts are shown in Table 1. UAEFD-TT extracts had the highest total catechins (341.38 ± 0.11 mg/g extract), while UAESD-MT extracts had the lowest (147.13 ± 0.36 mg/g extract). ECG was the most abundant catechin in UAEFD-TT and UAESD-TT extracts (109.63 ± 0.15 and 99.50 ± 0.00 mg/g extract, respectively), whereas CG (0.93 ± 0.05 mg/g extract and 0.47 ± 0.12 mg/g extract) and GC (5.90 ± 0.00 mg/g extract and 5.22 ± 0.01 mg/g extract) were the least abundant in these extracts, respectively. GCG and CG were not detected in the UAEFD-MT and UAESD-MT extracts. However, the CG content among UAESD-TT, UAEFD-ML, and UAESD-ML extracts was not significantly different (*p* > 0.05) (Table 1). The total catechins can be arranged according to their amounts in the following order: UAEFD-TT > UAESD-TT > UAEFD-ML > UAESD-ML > UAEFD-MT > UAESD-MT.

### 3.2. TPC and TFC of the Thai Assam Tea Extracts

The TPC and TFC of the extracts are shown in Table 2. Consistent with the results of catechin contents, the TFC and TPC in UAEFD-TT were higher than those in the other extracts (456.78 ± 4.31 mg GAE/g extract and 333.98 ± 0.83 mg QE/g extract, respectively), suggesting extraction of TT samples using UAE followed by FD increases the yield of the phenolic and flavonoid compounds in the Thai Assam tea extract.

### 3.3. Total Caffeine Content (CC) of the Thai Assam Tea Extracts

The CC of the extracts is presented in Table 2. The results showed that the CC in UAEFD-TT samples was the highest (93.20 ± 0.36 mg CF/g extract), whereas that in UAEFD-MT was the lowest (43.00 ± 0.56 mg CF/g extract). The CC content in samples extracted using UAE, followed by FD extracted from TT, was greater than those extracted from ML and MT; however, using the SD method, CC obtained from TT and ML did not show a significant difference (*p* > 0.05). The amounts of CC can be arranged as follows: UAEFD-TT > UAESD-TT > UAEFD-ML = UAESD-ML = UAESD-MT > UAEFD-MT.

### 3.4. Free Radical Scavenging and Ferrous Ion Chelating Activities of the Thai Assam Tea Extracts

The antioxidant activities of the extracts were assessed using DPPH, ABTS, and FRAP assays. The DPPH and ABTS assays revealed that UAEFD-TT exhibited strong free radical scavenging ability with IC_50_ values of 1.31 ± 0.02 *μ*g/mL for DPPH and 7.51 ± 0.03 *μ*g/mL for ABTS compared to the positive control (4.78 ± 0.08 *μ*g/mL of DPPH and 7.36 ± 0.10 *μ*g/mL of ABTS). FRAP assay revealed similar results and UAEFD-TT exhibited remarkably strong ferrous ion chelating ability (324.54 ± 3.33 *μ*M FeSO_4_/mg extract) than the remaining extracts (Table 3).

## 4. Discussion

In the present study, we investigated the effects of the drying method following UAE on the catechin profiles and antioxidant activities of extracts obtained from the leaves of Thai Assam tea at different maturity stages **(**TT, ML, and MT). The findings demonstrated that UAEFD-TT had the highest catechin content and CC. These extracts also exhibited the highest TPC and TFC and demonstrated the strongest antioxidant activity. The findings suggest that UAE, followed by FD, is an efficient method for extracting antioxidant compounds, and TT is the suitable stage to obtain extracts with enhanced quality.

HPLC analysis revealed high catechin and CCs in TT extracts, followed by ML and MT extracts obtained using UAE and different drying methods. However, FD increased the efficiency of catechins' yield in the extracts compared to SD [63]. Furthermore, in TT and ML extracts, the levels of EGCC and EGC were higher than those in the MT extracts, whereas GCG and CG were not identified in MT extracts. These findings of this study are consistent with those of a previous study, which showed markedly decreased EGCG content in ML, while EGC content was increased markedly in TT and ML [64]. Previous studies reported that the catechin and CCs depend on the stages of tea leaves with TT containing high EGCG, EGC, ECG, EC, and CF [65–67]. Similar to our findings, GC and GCG contents were the lowest or could not be detected in MT samples [68]. Moreover, the amount of bioactive compounds in tea extracts depends on seasonal variation, harvest method, leaf variety, and genetic variation [7, 64, 68].

UAE can enhance the mass-transfer rate of bioactive compounds during extraction from plant tissues [69], and the use of solvents increases catechin extraction efficiency and reduces extraction time [70]. Ultrasonic extraction is well established. The cell walls in the leaves can be destroyed by the action of the burrows. This resulted in increased extraction yield and reduced extraction solvent [29, 71]. Furthermore, previous studies have reported that a high yield of catechins was attained using 50% ethanol as the extraction solvent [70, 71]. This could be because the solvents increase the surface area under contact between the solvent and cell matrix, leading to increased efficiency of extraction [69]. The frequency, temperature, and power of the ultrasound should also influence the efficiency of the extraction method. The ultrasonic power of 150 W, ultrasound frequency of 37 kHz, and controlled solvent temperature of 30°C were used in our study. These were within the ranges of published optimal conditions for extractions of catechins in green tea, and 20–40 kHz frequency, 28°C–60°C temperature, and 50–461 W power have been shown to improve the extraction efficacy of total catechins in green tea [69, 72]. SD presents advantages such as cheap large-scale production, ease of change in operating conditions, and availability at both laboratory and industrial scales [40]. However, FD is effective in preserving the nutritional contents of powdered products [73]. In this study, we found that FD had a higher efficiency in recovering catechin contents than SD. This could be because low temperatures ensure the better preservation of bioactive compounds. Phenolic compounds are a type of polyphenol that are classified as tannins, propanoids, and flavonoids. Phenolic compounds are powerful chain-breaking antioxidants that may directly contribute to antioxidative activity [74, 75]. These compounds are important constituents of plants, and their radical scavenging ability is attributed to their hydroxyl groups [76]. DPPH and ABTS assays indicate radical scavenging activities, while FRAP assay presents the ability to reduce the ferric ions of the plant extracts [77]. In this study, the results showed that extracts with high TPC and TFC had high antioxidant activities. Consistent with our findings, strong correlations have been reported between the antioxidant capacities and TPC and TFC of the Thai Assam tea extract [78, 79]. These results indicate that the antioxidant activities of the plant extracts are most likely attributed to their polyphenol contents [80]. Previous studies reported that flavonoids have antioxidant, anti-inflammation, and antiarteriosclerotic activities [81]. Moreover, tea flavonoids have high antioxidant and radical scavenging activities [11, 39, 45, 52, 56, 82].

The findings of this study showed that the TT extracts had a higher TPC than the ML and MT extracts. In addition, UAEFD-TT exhibited a significantly higher antioxidant activity and polyphenol content **(**TPC and TFC**)**. Moreover, the TPC and TFC of Thai Assam tea extract were markedly higher than those reported in previous studies **(**TPC 391.48 ± 1.16 mg GAE/g extract and TFC 195.02 ± 13.14 mg QE/g extract) [54, 83]. The strong antioxidant activity of UAEFD-TT can be related to its high catechin contents. Furthermore, our findings also showed that the polyphenolic components and antioxidant values decreased with increasing maturity of leaves, which could be attributed to the morphological changes in leaves with age and unique chemical compound transportation within the plant [84]. Furthermore, extraction with aqueous methanol has been reported to contribute to higher antioxidant activity than pure methanol and hot water extraction [85, 86]. Our study is consistent with the study by Rafique et al. [87], who reported that the radical scavenger percentage of 50% ethanol tea extract (77.15%) was similar to that of the 80% ethanol solvent extract (77.70%) [87]. Our results, consistent with a previous report, suggested that different solvents with different polarities also influence the efficiency of the antioxidant activity of tea extract [88–90]. Taken together, we demonstrated that among the different tea preparations, UAEFD-TT had the highest antioxidant effects, catechin contents, TPC, and TFC. The information from this study may be beneficial to the development of Thai Assam tea for health promotion.

## 5. Conclusion

In conclusion, the present study evaluated the efficiency of SD and FD methods following UAE on the yield of catechins in the extracts obtained from Thai Assam tea leaves at different maturity levels (TT, ML, and MT). The results showed that UAEFD-TT had higher catechins, TPC, TFC, CC, and antioxidant activities compared to ML and MT leaf extracts obtained using the same method. This study highlights the importance of UAE, drying methods, and solvent choice in obtaining tea extracts, which contributes to the determination of the quality of Thai Assam tea cultivars. The present study can serve as a springboard to further improvement or development of Thai Assam tea extract, which can be considered as a herbal supplement.

## Figures and Tables

**Table 1:** Catechin contents in Thai Assam tea extracts.

Catechins (mg/g extract)	UAEFD-TT	UAESD-TT	UAEFD-ML	UAESD-ML	UAEFD-MT	UAESD-MT
GC	5.90 ± 0.00^a^	5.22 ± 0.01^b^	4.49 ± 0.01^c^	4.42 ± 0.02^d^	3.91 ± 0.01^e^	3.55 ± 0.06^f^
EGC	42.25 ± 0.09^a^	37.21 ± 0.01^b^	33.64 ± 0.12^c^	32.95 ± 0.36^d^	23.51 ± 0.17^e^	20.57 ± 0.09^f^
C	28.65 ± 0.05^a^	23.41 ± 0.01^e^	23.97 ± 0.02^d^	23.20 ± 0.00^f^	27.89 ± 0.06^b^	27.13 ± 0.02^c^
EC	59.32 ± 0.03^a^	36.40 ± 0.00^b^	34.91 ± 0.07^c^	34.12 ± 0.06^d^	22.89 ± 0.06^e^	20.39 ± 0.10^f^
EGCG	93.40 ± 0.17^a^	86.32 ± 0.01^b^	81.20 ± 0.49^c^	80.93 ± 0.06^d^	27.32 ± 0.01^e^	22.49 ± 0.06^f^
GCG	1.29 ± 0.04^a^	0.81 ± 0.01^b^	0.71 ± 0.03^c^	0.64 ± 0.06^d^	ND	ND
ECG	109.63 ± 0.15^a^	99.50 ± 0.00^b^	94.90 ± 0.07^c^	94.09 ± 0.06^d^	57.22 ± 0.01^e^	53.00 ± 0.15^f^
CG	0.93 ± 0.05^a^	0.47 ± 0.12^b^	0.48 ± 0.01^b^	0.41 ± 0.00^b^	ND	ND
**Total catechins**	341.38 ± 0.11^a^	289.34 ± 0.13^b^	274.31 ± 0.74^c^	270.75 ± 0.27^d^	162.74 ± 0.11^e^	147.13 ± 0.36^f^

*Note:* Each value is expressed as mean ± standard deviation.

Abbreviation: ND, not detected.

^a–f^Values in the same row with different superscripts are significantly different (*p* < 0.05).

**Table 2:** TPC, TFC, and CC of Thai Assam tea extracts.

Samples	TPC (mg GAE/g extract)	TFC (mg QE/g extract)	CC (mg CF/g extract)
UAEFD-TT	456.78 ± 4.31^a^	333.98 ± 0.83^a^	93.20 ± 0.36^a^
UAESD-TT	434.98 ± 3.33^b^	309.69 ± 0.60^b^	79.33 ± 0.31^b^
UAEFD-ML	394.70 ± 2.12^c^	247.84 ± 0.82^c^	73.03 ± 0.15^c^
UAESD-ML	334.82 ± 1.16^d^	240.91 ± 0.42^d^	72.80 ± 0.26^c^
UAEFD-MT	313.32 ± 2.45^e^	190.03 ± 0.62^e^	43.00 ± 0.56^d^
UAESD-MT	247.04 ± 2.57^f^	168.76 ± 0.46^f^	72.53 ± 0.25^c^

*Note:* Each value is expressed as mean ± standard deviation (*n* = 3).

^a–f^Values in the same column with different superscripts are significantly different (*p* < 0.05).

**Table 3:** Effect of Thai Assam tea extracts on antioxidant activity.

Samples	DPPH IC_50_ (*μ*g/mL)	ABTS IC_50_ (*μ*g/mL)	FRAP (*μ*M FeSO_4_/mg extract)
UAEFD-TT	1.31 ± 0.02^e^	7.51 ± 0.03^f^	324.54 ± 3.33^a^
UAESD-TT	1.81 ± 0.02^d^	8.56 ± 0.04^e^	250.64 ± 1.80^b^
UAEFD-ML	5.58 ± 0.30^a^	10.57 ± 0.02^c^	132.57 ± 2.33^c^
UAESD-ML	2.40 ± 0.02^c^	9.22 ± 0.02^d^	125.03 ± 3.07^d^
UAEFD-MT	4.59 ± 0.01^b^	12.30 ± 0.05^b^	115.98 ± 1.64^e^
UAESD-MT	4.73 ± 0.02^b^	13.90 ± 0.05^a^	102.20 ± 2.43^f^
EGCG	4.78 ± 0.08^b^	7.36 ± 0.10^f^	—

*Note:* Each value is expressed as mean ± standard deviation (*n* = 3).

^a–f^Values in the same row with different superscripts are significantly different (*p* < 0.05). Values followed by the same letter(s) are not significantly different (*p* > 0.05) according to Duncan's multiple range test.

## Data Availability

The data used to support the findings of this study are available in the supporting information of this article.

## Nomenclature

HPLC: High-performance liquid performance chromatography
UAE: Ultrasound-assisted extraction
FD: Freeze drying
SD: Spray drying
TT: Top leaves
ML: Middle leaves
MT: Mature leaves
DPPH: 2,2-Diphenyl-1-picrylhydrazyl
ABTS: 2,2′-Azino-bis (3-ethylbenzothiazoline-6-sulfonic acid)
FRAP: Ferric-reducing antioxidant power
EGCG: (−)-Epigallocatechin gallate
EGC: (−)-Epigallocatechin
ECG: (−)-Epicatechin gallate
EC: (−)-Epicatechin
GC: Gallocatechin
C: Catechin
CF: Caffeine
GCG: Gallocatechin gallate
CG: Catechin gallate
TFA: Trifluoroacetic acid
TPTZ: 2,4,6-Tris(2-pyridyl)-s-triazine
TPC: Total phenolic content
TFC: Total flavonoid content
CC: Caffeine content
mg GAE/g extract: Milligrams of gallic acid equivalent per gram extract
mg QE/ g extract: Milligrams of quercetin equivalent per gram extract

## Supporting Information

Supporting information includes Table S1: Weight of the Thai Assam tea extracts. Table S2: Retention time of catechins and caffeine from Thai Assam tea extract.
