# Supplementary material for: Effect of Ultrasound-Assisted Extraction and Drying Methods on Bioactive Compounds, Phenolic Composition, and Antioxidant Activity of Assam Tea Cultivar (Camellia sinensis var. assamica) Cultivated in Thailand
Source: Adv Pharmacol Pharm Sci. 2024 Oct 30;2024:5772961. doi: 10.1155/2024/5772961 (PMC11540878; doi:10.1155/2024/5772961)
Supplement: Supporting Information — Additional supporting information can be found online in the Supporting Information section. [file 5772961.f1.docx]

**Table S1.** Weight of the Thai Assam tea extracts.

| **Samples** | **Weight of dried**  **tea leaves (g)** | **Weight of crude**  **extract (g)** | **Weight after drying (g)** | **%Yield** |
| --- | --- | --- | --- | --- |
| UAEFD-TT | 100.53 ± 0.97 | 29.94 ± 0.88 | 11.18 ± 0.63 | 11.12 ± 0.32 |
| UAESD-TT | 100.23 ± 0.18 | 30.05 ± 0.88 | 14.51 ± 0.95 | 14.47 ± 0.67 |
| UAEFD-ML | 100.62 ± 0.10 | 30.22 ± 1.22 | 11.80 ± 0.04 | 11.78 ± 1.14 |
| UAESD-ML | 100.36 ± 0.81 | 29.69 ± 0.47 | 14.75 ± 0.11 | 14.69 ± 0.70 |
| UAEFD-MT | 100.92 ± 1.02 | 30.40 ± 0.98 | 11.93 ± 0.30 | 11.82 ± 1.01 |
| UAESD-MT | 100.58 ± 1.05 | 29.50 ± 0.59 | 15.11 ± 0.09 | 15.02 ± 0.44 |

Each value is expressed as mean ± standard deviation from three independent samples.

**Table S2.** Retention time of catechins and caffeine from Thai Assam tea extract.

Each value is expressed as mean ± standard deviation. GC; Gallocatechin, EGC; Epigallocatechin, C; Catechin, EC; Epicatechin, Epigallocatechin gallate; EGCG, CF; Caffeine, ECG; Epicatechin gallate, CG; Catechin gallate.

| Samples | Retention Time (min) | | | | | | | | |
| --- | --- | --- | --- | --- | --- | --- | --- | --- | --- |
|  | **GC** | **EGC** | **C** | **EC** | **EGCG** | **CF** | **GCG** | **ECG** | **CG** |
| Standard Mix | 1.271 ± 0.001 | 1.668 ± 0.001 | 1.937 ± 0.001 | 2.563 ± 0.001 | 2.838 ± 0.001 | 3.465 ± 0.001 | 3.906 ± 0.001 | 4.631 ± 0.001 | 6.221 ± 0.001 |
| UAEFD-TT | 1.258 ± 0.001 | 1.647 ± 0.001 | 1.897 ± 0.001 | 2.517 ± 0.001 | 2.844 ± 0.001 | 3.463 ± 0.001 | 4.003 ± 0.002 | 4.615 ± 0.002 | 6.240 ± 0.006 |
| UAEFD-MT | 1.255 ± 0.001 | 1.646 ± 0.001 | 1.896 ± 0.001 | 2.514 ± 0.002 | 2.843 ± 0.002 | 3.466 ± 0.002 | 3.990 ± 0.008 | 4.612 ± 0.004 | 6.229 ± 0.002 |
| UAEFD-ML | 1.263 ± 0.004 | 1.652 ± 0.004 | 1.903 ± 0.004 | 2.522 ± 0.004 | 2.850 ± 0.004 | 3.468 ± 0.004 | 4.002 ± 0.002 | 4.619 ± 0.004 | 6.242 ± 0.004 |
| UAESD-TT | 1.260 ± 0.004 | 1.648 ± 0.004 | 1.898 ± 0.004 | 2.518 ± 0.003 | 2.845 ± 0.003 | 3.464 ± 0.003 | 4.004 ± 0.003 | 4.615 ± 0.003 | 6.243 ± 0.004 |
| UAESD-MT | 1.260 ± 0.003 | 1.649 ± 0.003 | 1.899 ± 0.003 | 2.515 ± 0.003 | 2.845 ± 0.004 | 3.467 ± 0.003 | 3.988 ± 0.006 | 4.605 ± 0.006 | 6.206 ± 0.004 |
| UAESD-ML | 1.268 ± 0.008 | 1.655 ± 0.005 | 1.903 ± 0.002 | 2.520 ± 0.002 | 2.845 ± 0.005 | 3.464 ± 0.005 | 3.995 ± 0.014 | 4.606 ± 0.016 | 6.224 ± 0.014 |
